# Patient-Reported Experiences With Viewing and Understanding Test Results in Patient Portals: Cross-Sectional Survey Analysis

**DOI:** 10.2196/94098

**Published:** 2026-06-12

**Authors:** Chelsea Richwine, Bryan Steitz, Jordan Everson

**Affiliations:** 1 Office of the National Coordinator for Health Information Technology, U.S. Department of Health and Human Services, 330 C St SW, Washington, DC, 20201, United States, 1 771-210-0823; 2 Department of Biomedical Informatics, Vanderbilt University Medical Center, Nashville, TN, United States; 3 Department of Family Medicine, Georgetown University, Washington, DC, United States

**Keywords:** personal health records, patient portal, patient engagement, electronic health records, health information technology, communication

## Abstract

**Background:**

21st Century Cures Act information blocking regulations led to many organizations altering policies to electronically release test results to patients immediately upon their availability.

**Objective:**

This study aims to understand the prevalence and variation in how often patients view test results in the patient portal before hearing from their health care provider (HCP), are given the option to decide how results are communicated, and understanding of results viewed before hearing from their HCP.

**Methods:**

In this cross-sectional analysis, we analyzed data from the 2024 Health Information National Trends Survey on US adults with complete information who received test results via patient portal (n=6045). We examined the share of all patients and “portal users” (accessed their portal in the last year) who viewed test results in their portal, viewed results before hearing from their HCP, and were given the option to decide how results were communicated. We examined patients’ understanding of results viewed before hearing from their HCP and compared it with their understanding of other health information. Finally, we examined differences in weighted proportions of outcomes by individual characteristics using survey-weighted linear regression. Differences were assessed using design-adjusted Wald tests.

**Results:**

In 2024, 70% of patients (92% of portal users) viewed results in their patient portal, and 58% (76% of portal users) viewed the results before hearing from their HCP. Overall, 28% of patients and 33% of portal users reported being given the option to decide whether they wanted to receive test results before hearing from their HCP. Only 66% of patients reported that they understood the results they viewed in their patient portal. Provider encouragement, higher education, and digital literacy were most strongly associated with higher rates of viewing test results overall and before hearing from their HCP (eg, 70% of those encouraged viewed results vs 24% not encouraged; *P*<.001). Associations with age and chronic condition were also observed but were weaker. While higher education and digital literacy, age, and provider encouragement were strongly associated with being given the option to decide how to receive results, only high digital literacy and provider encouragement were strongly, positively associated with understanding results viewed before hearing from their HCP (eg, 77% of patients with high digital literacy understood results compared to 53% of those with low digital literacy; *P*<.001).

**Conclusions:**

This study is the first to provide a national estimate of patients who access immediately released test results and whether patients are given the option to decide how their results are communicated to them. While most patients view results before discussing them with their HCP, rates varied by individual characteristics, and substantially fewer patients report being given the option to decide how results are communicated. Incorporating patient preferences in portal communications may empower patients while preserving immediate access.

## Introduction

Individuals have long had a right to access their protected health information [1]. In practice, potentially sensitive information like laboratory and imaging results were often not available to patients until health care providers (HCPs) had a chance to review, interpret, and follow up on findings [2]. In implementing the 21st Century Cures Act provision prohibiting information blocking, the US Department of Health and Human Services indicated that broad organizational policies that delay the release of health information to patients were likely to implicate information blocking, even if an HCP has not yet had a chance to review the result or personally inform the patient of the results before they can electronically access them [3,4]. Consequently, many organizations now immediately release test results to the patient portal, leading to more patients accessing their results before they are reviewed by the ordering HCP [5,6]. Some have expressed concerns that immediate release of results without additional context and interpretation from an HCP could cause patients to experience negative emotions [7,8]. However, studies conducted since the information blocking regulations became applicable on April 5, 2021, have shown that patients overwhelmingly prefer having immediate access to test results, even if it means viewing those results before discussing them with their HCP [9-11]. However, preferences for immediate release may depend on the severity or sensitivity of the results, and some patients may prefer to be contacted by their HCP shortly after receiving abnormal results [12-14].

While several studies have sought to understand patient preferences around viewing their immediately released results in the patient portal, it is unclear to what extent patient preference is considered by health care organizations when information is released in patient portals. For instance, whether patients were made aware they might receive results in their portal before their HCP could discuss them, and if they were given the option to decide whether they wanted results immediately released to their portal, held until their HCP released them, or alerted to new results upon release [15]. Further, little is known about whether patients who viewed test results in their portal before hearing from their HCP felt that they understood those results and what they meant for their care. Conversations during health care visits present an opportunity for HCPs to provide context to tests being ordered and help prepare patients on what to expect in terms of communication and follow-up. Test results provided through the portal may therefore omit helpful context.

This study is the first to provide a national estimate of the share of patients who access immediately released test results and sheds light on the extent to which patients are given the option to decide how their results are communicated to them. We used nationally representative survey data to examine (1) whether patients viewed test results in the patient portal before hearing from their HCP, (2) whether patients were given the option to decide how test results were communicated, and (3) whether they understood the results and their implications. To contextualize these findings, we compared patients’ understanding of test results to their general understanding of information in the patient portal and HCP explanations during health care visits and examined differences by digital health literacy and clinical needs. This work provides national evidence on how patients engage with immediately available test results, their communication preferences, and their preparedness to interpret results before consulting their HCP.

## Methods

### Inclusion and Exclusion

Data for this cross-sectional survey study came from the Health Information National Trends Survey (HINTS) 7 (2024), a nationally representative survey of U.S. noninstitutionalized adults conducted by the National Cancer Institute that tracks individuals’ understanding and use of health-related information and health information technology. The analytic sample used for this secondary analysis of the HINTS was limited to 6335 respondents who had test results in the past year, as determined by responses to question E10 on HINTS 7. It excluded respondents with missing data for test results-related questions in E3, E5, E9, and E11 (<1% across all measures), resulting in a total sample of 6045 patients with recent test results.

### Participant Characteristics

The total study population included 6045 patients (mean age, 50.07, SE 0.24 years), for whom self-reported level of education was as follows: 344 (6%) had less than high school education, 935 (20%) had high school education, 1791 (39%) had some college, and 2975 (35%) had a college education (Table S1 in Multimedia Appendix 1). Quartiles of digital literacy were as follows: 2280 (33%) in the bottom quartile (lowest digital literacy), 813 (13%) in the second quartile, 1836 (33%) in the third quartile, and 1103 (21%) in the top quartile (highest digital literacy). For health status: 3994 (62%) reported at least one chronic condition (diabetes, high blood pressure, heart condition, lung disease, or depression), and 302 (3%) had a recent cancer diagnosis. 4558 (76%) respondents reported being encouraged by their HCP to use their portal.

### Sampling Procedures

The survey was fielded as both a paper and web-based survey from March 25, 2024, to September 16, 2024. The sample design consisted of 2 stages in which a stratified sample of addresses was first selected from a file of residential addresses, and then one adult was selected within each household. The sampling frame consisted of a database of addresses used by Marketing Systems Group to provide a random sample of addresses. The sampling frame of addresses was grouped into 4 explicit sampling strata by racial or ethnic minority and rural status. Both survey modes (paper and web) were offered in English or Spanish. Full details on HINTS 7 sample selection, data collection, and management are available in the HINTS 7 methodology report [16].

### Sample Size, Power, and Precision

The total number of addresses selected for HINTS 7 was 36,000 (by strata: 25,300 from high minority urban, 1950 from high minority rural, 6100 from low minority urban, and 2650 from the low minority rural), which based on historical HINTS response rates, was the sample size needed to achieve the target number of 7000 complete questionnaires. The HINTS over-sampled high-minority and rural strata to increase the precision of estimates for racial or ethnic minority and rural subpopulations. Ultimately, complete questionnaires were collected from 7278 respondents (27.31% response rate).

### Ethical Considerations

The HINTS 7 general population survey was designated “exempt research” under 45 CFR 46.104. HINTS 7 also received a “Not Human Subjects Research” determination from the NIH (National Institutes of Health) Office of IRB Operations (IRBID: IRB002042). Compensation type and amount for human participants research are therefore not applicable to this study. To protect the privacy of respondents, HINTS data are deidentified and do not contain any geographical indicators. No identification of individual participants or users in any images of the manuscript or supplementary material is possible. Publicly available data are available at the aggregate level only. This study was deemed exempt from institutional review board review as it involved secondary analysis of publicly available, deidentified data that does not involve human participants [17].

### Measures and Covariates

#### Outcomes

We defined 4 binary outcomes of interest related to patients’ experiences receiving communication about test results, viewing results in their online medical record or patient portal, and understanding those results before discussing them with their HCP. All survey questions asked about actions occurring in the past 12 months.

View results. The first outcome was derived from question E5 on HINTS 7 and indicated patients who reported using their portal to view test results (equal to ‘1’ if “Yes,” equal to ‘0’ if “No” or “Not applicable” (ie, did not access portal in the past 12 months).

View results before hearing from HCP*.* The second outcome was derived from question E10 on HINTS 7 and indicated patients who reported using their portal to view test results *before* hearing about the result from their HCP (equal to "1" if “Yes,” equal to ‘0’ if “No”). Respondents who did not have any medical tests in the past 12 months were excluded from the study.

Option to decide. The third outcome was derived from question E9 on the HINTS and indicated patients who reported being given the option to decide whether they wanted to receive test results before their HCP could discuss the results with them (equal to "1" if “Yes,” equal to ‘0’ if “No” or “Don’t know”).

Understand results before hearing from HCP. The fourth outcome was derived from question E11 on the HINTS. We converted the measure from a Likert scale into a binary variable that indicates patients who reported they understood “very well” or “well” what the test results showed and what they meant for their care (equal to "0" if “fairly well” or “poorly”). This question was only asked of individuals who indicated they viewed test results in their portal before hearing from their HCP.

The full list of survey questions is available on the HINTS 7 Survey Instrument on the HINTS website [18]. A mapping of all outcomes and measures to survey questions on HINTS 7 is available in Table S2 in Multimedia Appendix 1.

#### Comparable Measures Related to Understanding Information

Individuals’ understanding of test results they viewed in their portal before hearing from their HCP was compared to patients’ general understanding of information in their portal and HCP explanations of health information communicated during a health care visit. The measure of general understanding of information in the portal was derived from question E6 on HINTS 7 and defined as a binary variable indicating patients who reported it was “very” or “somewhat” easy to understand health information in their online medical record or patient portal (equal to “0” if “somewhat” or “very” difficult). The measure of HCPs explaining information clearly during health care visits was derived from question C4e. on the HINTS and defined as a binary variable indicating patients who reported their HCPs “always” or “usually” explain things in a way they can understand (equal to “0” if “sometimes” or “never”).

#### Individual Characteristics

We examined differences in outcomes across characteristics that may influence individuals’ comfort or need for using that technology including age, education, digital literacy, and having a recent cancer diagnosis in the last 5 years or a chronic condition (including diabetes, high blood pressure, heart condition, lung disease, or depression), as these individuals have been shown to be more frequent users of patient portals [19]. We also examined outcomes among individuals who were encouraged by an HCP to use the patient portal, which has been shown to influence patient portal access and use [20]. We created a digital literacy index from 3 correlated measures derived from question B5 on HINTS 7 that were intended to capture digital literacy by asking patients about the extent to which they agree or disagree with statements about their comfort with technology and digital tools (a. “I find learning how to use new technology frustrating,” “b. I can use applications/programs on my cell phone or computer without asking someone for help,” and “c. I have the skills to find the health information I need on the Internet”). The index was created by taking the average across the 3 self-reported measures. We then used quartiles of the digital literacy index to capture the range of digital literacy, where those in the bottom quartiles (closest to 1) have lower digital literacy (ie, more difficulty with technology, less comfort with digital tools) and those in the top quartiles (closer to 4) have the highest digital literacy.

### Statistical Analysis

Leveraging our analytic sample, we first compared the proportions of individuals who viewed test results in their patient portal, viewed results in their portal *before* hearing from their HCP, and reported being given the option to decide whether they wanted to receive results before hearing from their HCP. We examined rates among all patients with recent results and among those who accessed their patient portal in the past 12 months (“portal users”). We then examined patients’ understanding of test results they viewed in their portal before hearing from their HCP, and how this compared to their general understanding of information in their portal and HCP explanations of health information. Finally, we examined differences in the weighted proportion of patients who viewed results in their portal (overall and before hearing from their HCP), reported being given the option to decide how to receive results, and understood the results they viewed before discussing with their HCP by demographic and health characteristics. Differences between groups were assessed using survey-weighted linear regression with statistical significance evaluated using design-adjusted Wald tests. All analyses used survey weighting procedures with jackknife replicate weights, which were designed to correct for nonresponse bias and under-sampling as well as to enable valid inferences from the study sample to the population. See the HINTS 7 Methodology report for more details on sample weights [16]. Data analyses were performed in January to March 2025 using Stata/SE (version 15.1; StataCorp).

## Results

**Figure 1.**
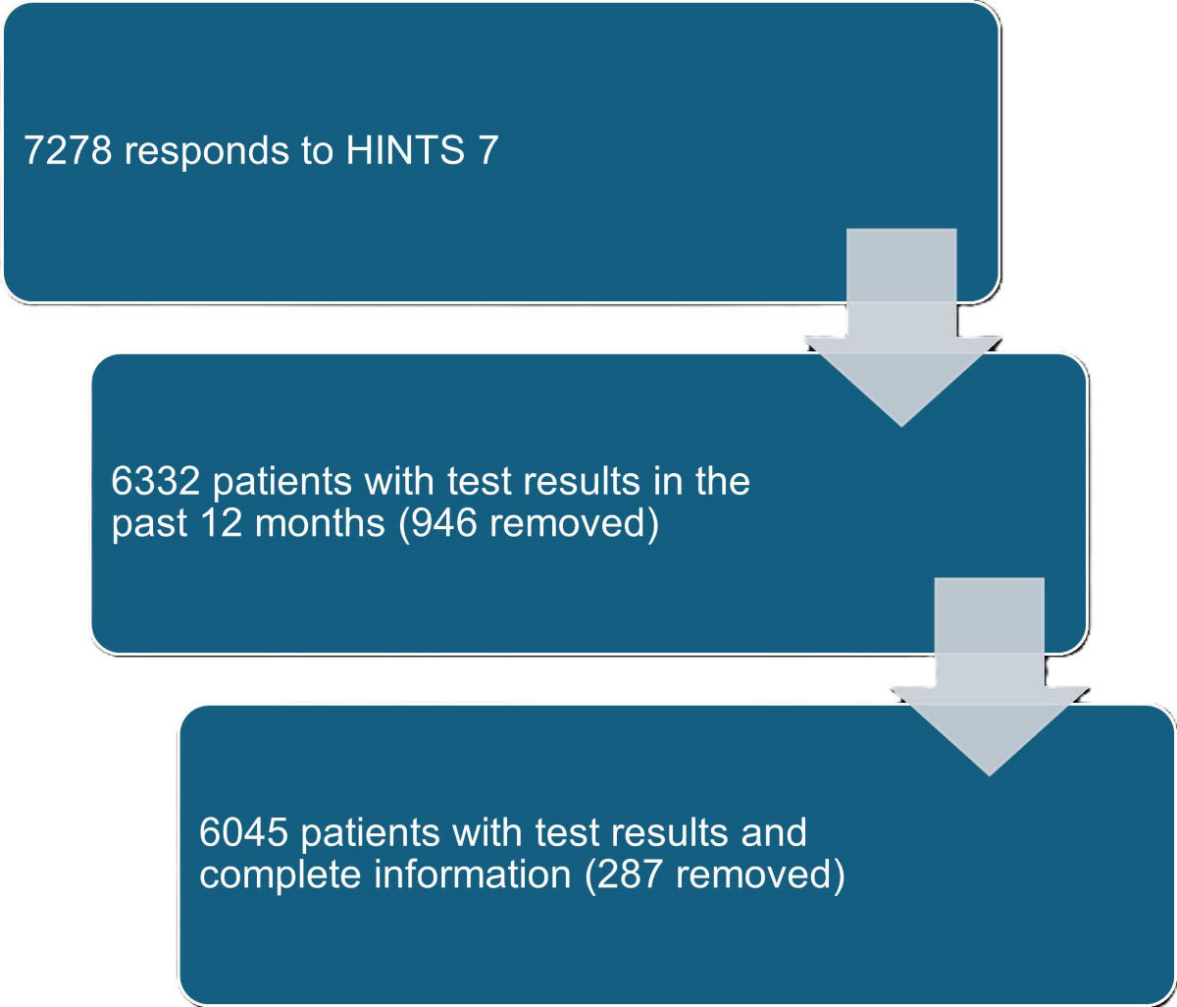
Creation of analytic data set from Health Information National Trends Survey 7. HINTS 7: Health Information National Trends Survey 7.

The selection of the analytic sample for this study is shown in Figure 1 below. 7278 individuals responded to HINTS 7, 6332 individuals or “patients” had test results in the past 12 months, and 6045 patients with test results had complete information across measures. Data missingness was less than 1% across all measures.

In 2024, most patients who received test results reported viewing those results in their patient portal (70% overall, 92.4% among portal users) and viewed the results in their portal before hearing from their HCP (57.6% overall and 76% of portal users; Table 1). About one-quarter of patients (28%) and one-third of portal users (33.3%) reported being given the option to decide whether they wanted to receive test results before their HCP could discuss the results with them.

**Table 1.** Percent of patients with test results who viewed results in their patient portal overall and before hearing from their HCP, and the share of patients who reported they were given the option to decide whether they wanted to receive results before hearing from their HCP, 2024^a^.

Response	All individuals (n=6045)			Patient portal users (n=4485)		
	(1) View results, n (%) [95% CI]	(2) View results before hearing from HCP^b^, n (%) [95% CI]	(3) Option to Decide, n (%) [95% CI]	(4) View results, n (%) [95% CI]	(5) View results before hearing from HCP, n (%) [95% CI]	(6) Option to decide, n (%) [95% CI]
Yes	4172 (70) [68.2-71.8]	3432 (57.6) [28.2-31.8]	1768 (28) [26.2-29.9]	4172 (92.4) [90.9-93.7]	3432 (76) [73.9-77.8]	1545 (33.3) [31.2-35.5]
No	1873 (30) [28.2-31.8]	2613 (42.4) [40.7-44.2]	3214 (53.1) [50.8-55.4]	313 (7.6) [6.3-9.1]	1053 (24.1) [22.2-26.1]	2146 (48.2) [45.9-50.4]
Don’t know	—^c^	—	1063 (18.9) [16.9-21]	—	—	794 (18.5) [16.5-20.7]

a In column (1), the "No" category includes respondents who did not use their portal to view test results as well as those who did not access their patient portal in the past 12 months. In columns (4)-(6), patient portal users include individuals who reported accessing their patient portal or online medical record at least once in the past year. All percentages are weighted. 95% CI for point estimates reported in brackets.

b HCP: health care provider.

c not applicable.

**Figure 2.**
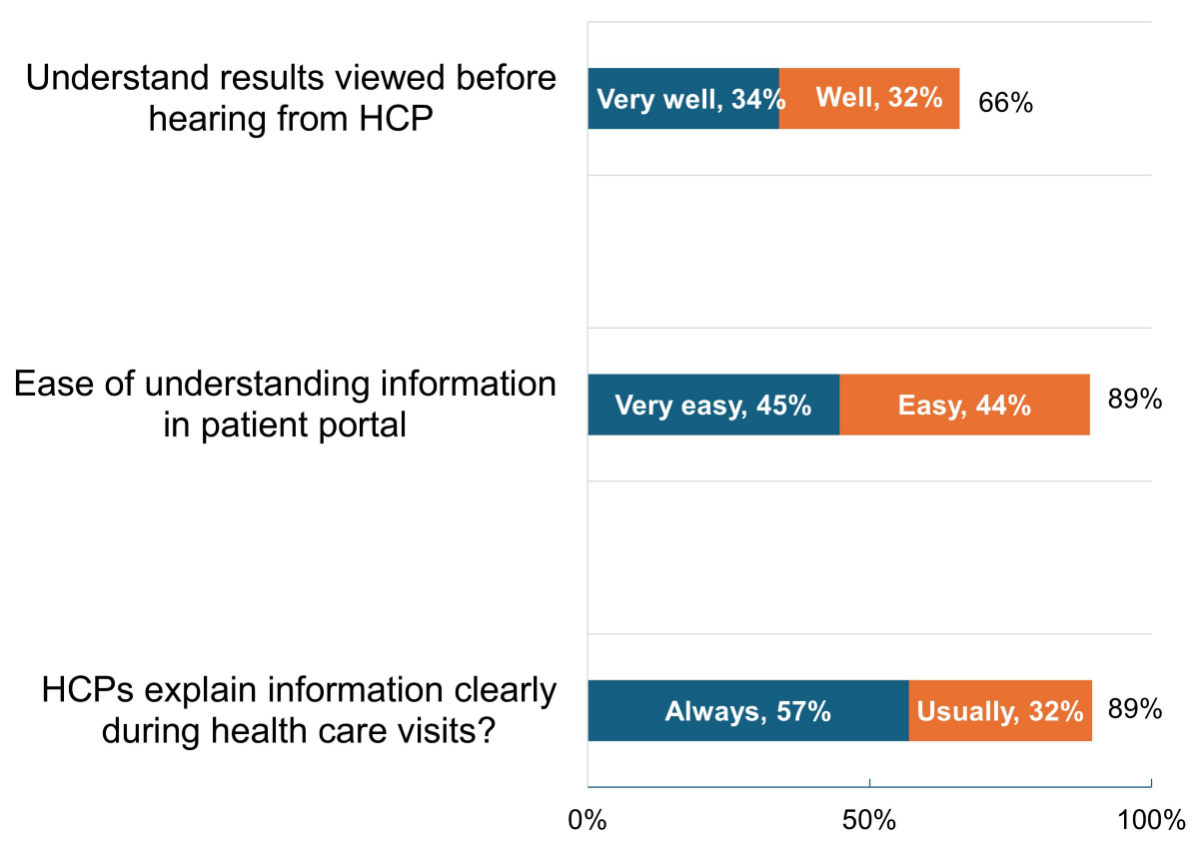
Patients’ understanding of test results compared to information in the patient portal and health care provider explanations, 2024. Notes: “Understand results viewed before hearing from HCP” was only asked of individuals who viewed test results in their patient portal before hearing from their health care provider (n=3432) and indicates patients who reported they understood the results very well or well. “Ease of understanding information in patient portal” was only asked of patient portal users (n=4485) and indicates patients who reported it was very or somewhat easy to understand health information in their online medical record or patient portal. “HCPs explain information clearly during health care visits?” was only asked of individuals who had a health care visit in the past year (n=5553) and indicates patients who reported their health care providers always or usually explains things in a way they can understand. All percentages are weighted. Full output reported in Table S3 in Multimedia Appendix 1. HCP: health care provider.

Two-thirds of patients (66%) reported that they understood test results they viewed in their patient portal before hearing from their HCP “well” (32%, 95% CI 29%‐35.1%) or “very well” (34%, 95% CI 30.9%‐37.2%) and what those results meant for their care. In contrast, 89% of patients in total indicated it was “somewhat” (44.4%, 95% CI 41.6%‐47.3%) or “very” (44.7%, 95% CI 42%‐47.3%) easy to understand information in their patient portal and that HCPs “usually” (32.4%, 95% CI 30%‐34.9%) or “always” (57%, 95% CI 54.5%‐59.5%) explained information clearly during a health care visit (Figure 2, Table S3 in Multimedia Appendix 1).

Patients and portal users aged 35‐74 and those with higher education were significantly more likely to view test results in their portal overall and before hearing from their HCP (eg, 71.1%, 95% CI 68.4%‐73.7% of college-educated patients viewed results before hearing from their HCP vs 30.8%, 95% CI 23.6%‐38.1% of those with less than high school education, *P*<.001; Tables2  3). However, confidence intervals around age estimates were wider, indicating less precise estimates. Patients and portal users with higher levels of digital literacy were also significantly more likely to view test results in their portal overall and before hearing from their HCP compared to patients with lower digital literacy (eg, 68.7%, 95% CI 63.5%‐73.9% vs 46.1%, 95% CI 41.7%‐50.5% viewed results before hearing from their HCP, *P*<.001). See Table S4 in Multimedia Appendix 1 for the share of patients who viewed test results in their portal across digital literacy index measures.

**Table 2.** Differences in all patients’ and portal users’ reports of viewing test results in their patient portal, by individual characteristics, 2024^a^.

Characteristics	Participants, n	All patients (n=6045)				Portal users (n=4485)			
		Weighted % (95% CI)			*P* value	Weighted % (95% CI)			*P* value
All individuals	4172	70			—^b^	92.4			—
Age (years)									
18‐34 (ref)	662	64.9 (59.8-70)			—	87.1 (81.7-92.4)			—
35‐49	950	78.8 (75.1-82.4)			<.001	95.2 (93.1-97.3)			.009
50‐64	1119	71.1 (68.1-74.2)			.06	93.7 (91.7-95.6)			.03
65‐74	920	69.9 (66-73.9)			.12	93.8 (90.7-96.9)			.048
75+	521	55.3 (48.6-62.1)			.03	90.6 (86.9-94.2)			.29
Education									
Less than high school (ref)	119	35.9 (26.2-45.6)			—	71.8 (52.2-91.4)			—
High school	486	58.1 (52.5-63.7)			<.001	92.3 (88.6-96)			.05
Some college	1202	70.6 (67.4-73.9)			<.001	92.3 (89.5-95.1)			.047
College	2365	81.7 (79.4-84)			<.001	94.5 (93.2-95.8)			.03
Digital literacy									
1 (low) (ref)	1213	56.2 (52.1-60.3)			—	89.2 (85.8-92.6)			—
2	563	62.3 (55.9-68.8)			.15	86.3 (79.4-93.1)			.46
3	1504	80.2 (76.5-83.9)			<.001	95.1 (93.1-97.1)			.006
4 (high)	888	81.2 (76.8-85.5)			<.001	95.1 (92.8-97.4)			.008
Chronic condition									
Yes	2762	72.2 (69.8-74.5)			—	93.9 (92.8-95.1)			—
No	1358	66.6 (62.6-70.6)			.03	89.7 (86.1-93.3)			.04
Recent cancer diagnosis									
Yes	231	76.7 (67.6-85.9)			—	94 (87.4-100.5)			—
No	3755	70 (68-72.1)			.18	92.4 (90.9-93.9)			.65
Encouraged to use patient portal									
Yes	3763	82.7 (81-84.4)			—	93.7 (92.3-95.1)			—
No	398	26.2 (22.4-29.9)			<.001	80 (74-86.1)			<.001

a Differences between groups were assessed using survey-weighted linear regression; statistical significance was evaluated using design-adjusted Wald tests. The numerator, n, is the same for all patients and portal users since, by definition, only portal users can view test results in their patient portal.

b not available.

**Table 3.** Differences in all patients’ and portal users’ reports of viewing test results in their patient portal before hearing from their HCP, by individual characteristics, 2024^a^.

Characteristics	Participants, n	All patients (n=6045)				Portal users (n=4485)			
		Weighted % (95% CI)			*P* value	Weighted % (95% CI)		*P* value	
All individuals	3432	57.6 (reference)			—^b^	76 (reference)		—	
Age (years)									
18‐34 (ref)	524	53.9 (48.6-59.2)			—	68.6 (63.1-74.1)		—	
35‐49	794	67.3 (63.5-71.1)			<.001	78.6 (74.6-82.5)		.006	
50‐64	918	61.8 (58.6-65)			.02	77.9 (73.7-82)		.02	
65‐74	769	61.4 (58.2-64.6)			.02	80.6 (76.7-84.5)		.002	
75+	427	46.4 (39.8-52.9)			.08	72.3 (65.9-78.6)		.37	
Education									
Less than high school (ref)	93	30.8 (23.6-38.1)			—	53.9 (36.9-70.9)		—	
High school	395	50.4 (44.7-56.1)			<.001	77 (70.5-83.5)		.01	
Some college	960	59 (55.9-62.1)			<.001	73.7 (69.6-77.7)		.03	
College	1984	71.1 (68.4-73.7)			<.001	79.8 (77.4-82.2)		.003	
Digital literacy									
1 (low) (ref)	948	46.1 (41.7-50.5)			—	68.2 (63.1-73.3)		—	
2	478	58.9 (52.8-65)			.004	76.4 (69-83.8)		.07	
3	1262	68.9 (65.3-72.4)			<.001	80.1 (76.9-83.3)		.001	
4 (high)	741	68.7 (63.5-73.9)			<.001	78.4 (73.9-82.9)		.01	
Chronic condition									
Yes	2296	61.9 (59.3-64.5)			—	77.6 (74.6-80.6)		—	
No	1100	56.5 (52.4-60.7)			.07	73 (69-77)		.11	
Recent cancer diagnosis									
Yes	203	70.8 (61.5-80.2)			—	84 (75.8-92.1)		—	
No	3087	59.8 (57.9-61.6)			.03	75.8 (73.8-77.8)		.049	
Encouraged to use patient portal									
Yes	3123	70.1 (68.1-72.2)			—	77.6 (75.4-79.8)		—	
No	301	24.3 (20.9-27.7)			<.001	59.8 (54.2-65.4)		<.001	

a Differences between groups were assessed using survey-weighted linear regression; statistical significance was evaluated using design-adjusted Wald tests. The numerator, n, is the same for all patients and portal users since, by definition, only portal users can view test results in their patient portal.

b not available.

Patients with chronic conditions were more likely to view test results in their patient portal (72.2%, 95% CI 69.8%‐74.5% vs 66.6%, 95% CI 62.6%‐70.6% for all patients, *P*=.03; 93.9%, 95% CI 92.8%‐95.1% vs 89.7%, 95% CI 86.1%‐93.3% among portal users, *P*=.04). However, they were no more or less likely to view results before hearing from their HCP. Conversely, patients with a recent cancer diagnosis were not more likely to view results in their portal overall but were more likely to view results before hearing from their HCP (70.8%, 95% CI 61.5%‐80.2% vs 59.8%, 95% CI 57.9%‐61.6% for all patients, *P*=.03; 84%, 95% CI 75.8%‐92.1% vs 75.8%, 95% CI 73.8%‐77.8% among portal users, *P*=.049). However, this difference was only marginally significant, and estimates were less precise due to the small sample of individuals with a recent cancer diagnosis.

Provider encouragement was the most strongly associated with viewing test results in the portal overall (82.7%, 95% CI 81%‐84.4% vs 26.2%, 95% CI 22.4%‐29.9% for all patients, *P*<.001; 93.7%, 95% CI 92.3%‐95.1% vs 80%, 95% CI 74%‐86.1% among portal users, *P*<.001) and before hearing from their HCP (70.1%, 95% CI 68.1%‐72.2% vs 24.3%, 95% CI 20.9%‐27.7% for all patients *P*<.001; 77.6%, 95% CI 75.4%‐79.8% vs 59.8%, 95% CI 54.2%‐65.4% among portal users, *P*<.001).

Patients with higher levels of education were more likely to report being given the option to decide whether to receive test results before hearing from their HCP; however, among portal users, there was only a moderate, significant difference among those with a college education compared to those with less than high school education (34.8%, 95% CI 31.8%‐37.7% vs 24%, 95% CI 14.3%‐33.8%; *P*=.03; Table 4).

**Table 4.** Differences in all patients’ and portal users’ reports of being given the option to decide how to receive test results, by individual characteristics, 2024^a^.

Characteristics	All patients (n=6045)					Portal users (n=4485)				
	Patients, n	Weighted % (95% CI)			*P* value	Portal users, n	Weighted % (95% CI)			*P* value
All individuals	1768	28			—^b^	1545	33.3			—
Age (years)										
18‐34 (ref)	272	27.5 (23.2-31.8)			—	246	32.7 (27.8-37.6)			—
35‐49	378	29.3 (25.8-32.8)			.50	344	32.7 (28.7-36.7)			.99
50‐64	435	25.8 (21.9-29.7)			.56	380	31.5 (26.5-36.5)			.76
65‐74	431	34 (30.2-37.8)			.01	376	40.8 (36-45.6)			.01
75+	252	23.3 (18.8-27.7)			.19	199	31 (24.1-37.9)			.68
Education										
Less than high school (ref)	71	17.3 (12.1-22.4)			—	45	24 (14.3-33.8)			—
High school	238	25.8 (20.4-31.2)			.03	177	32.3 (26-38.5)			.16
Some college	514	27.3 (23.9-30.7)			.002	455	33.2 (29.1-37.3)			.08
College	945	31.7 (29.2-34.3)			<.001	868	34.8 (31.8-37.7)			.03
Digital literacy										
1 (low) (ref)	569	24.4 (21.5-27.2)			—	441	32.3 (28.1-36.5)			—
2	252	28.1 (23.4-32.8)			.19	222	33.6 (27.8-39.4)			.71
3	582	28.9 (25.5-32.2)			.03	546	32.8 (29.1-36.5)			.86
4 (high)	363	32.5 (27.6- 37.3)			.007	334	35.2 (29.7-40.7)			.42
Chronic condition										
Yes	1218	29.1 (26.6-31.6)			—	1050	33.7 (30.9-36.4)			—
No	530	25.9 (23-28.9)			.11	479	32.3 (28.6-36)			.57
Recent cancer diagnosis										
Yes	90	27.3 (18.6-35.9)			—	78	30.8 (20.5-41)			—
No	1606	28.1 (26.2-30.1)			.85	1400	33.4 (31.2-35.6)			.61
Encouraged to use patient portal										
Yes	1551	32.5 (30.4-34.6)			—	1435	34.6 (32.4-36.9)			—
No	208	12.3 (9.9-14.7)			<.001	105	21 (14.8-27.2)			<.001

a Differences between groups were assessed using survey-weighted linear regression; statistical significance was evaluated using design-adjusted Wald tests.

b not available.

Patients with high digital literacy were also more likely to report being given the option to decide how to receive results compared to those with low digital literacy (32.5%, 95% CI 27.6%‐37.3% vs 24.4%, 95% CI 21.5%‐27.2%; *P*=.007)—though there were no significant differences among portal users. See Table S4 in Multimedia Appendix 1 for the share of patients who reported being given the option to decide how to receive results, and understanding of those results, across digital literacy index measures. Patients who reported being encouraged by their HCP to use their patient portal were significantly more likely to report being given the option to decide how to receive results (32.5%, 95% CI 30.4%‐34.6% vs 12.3%, 95% CI 9.9%‐14.7% for all patients, *P*<.001; 34.6%, 95% CI 32.4%‐36.9% vs 21%, 95% CI 14.8%‐27.2% for portal users, *P*<.001).

Digital literacy and provider encouragement were the only factors that were significantly, positively associated with patients’ understanding of results viewed before hearing from their HCP. High digital literacy was most strongly associated with understanding of results compared to low digital literacy (77.4%, 95% CI 71.5%‐83.4% vs 53%, 95% CI 47.7%‐58.4%; *P*<.001; Table 5). Individuals encouraged to use their portal by their HCP were also more likely to indicate they understood results they viewed before hearing from their HCP (67%, 95% CI 63.9%‐70% vs 54%, 95% CI 42.3%‐65.6%; *P*=.03).

**Table 5.** Differences in understanding of test results among portal users who viewed results before hearing from their health care provider, by individual characteristics, 2024^a^.

Characteristics	Portal users who viewed results before hearing from HCP (n=3432)				
	Portal users, n	Weighted % (95% CI)			*P* value
All individuals	2251	65.9			—^b^
Age, years					
18‐34 (ref)	347	64.8 (56.8-72.9)			—
35‐49	547	66.7 (60.6-72.9)			.71
50‐64	603	68.6 (63.3-74)			.43
65‐74	496	62.7 (56.6-68.9)			.69
75+	258	61.2 (53.2-69.3)			.49
Education					
Less than high school (ref)	50	62.4 (46.5-78.3)			—
High school	246	65.3 (57.9-72.7)			.72
Some college	596	62.9 (57.4-68.3)			.95
College	1359	69.2 (65.6-72.7)			.41
Digital literacy					
1 (low) (ref)	513	53 (47.7-58.4)			—
2	302	59.9 (50.9-69)			.17
3	868	68.9 (64.5-73.2)			<.001
4 (high)	566	77.4 (71.5-83.4)			<.001
Chronic condition					
Yes	1472	64.4 (60.3-68.4)			—
No	756	68.7 (63.6-73.7)			.17
Recent cancer diagnosis					
Yes	130	66.6 (52.8-80.4)			—
No	2031	66 (62.6-69.4)			.94
Encouraged to use patient portal					
Yes	2077	67 (63.9-70)			—
No	169	54 (42.3-65.6)			.03

a Differences between groups were assessed using survey-weighted linear regression; statistical significance was evaluated using design-adjusted Wald tests.

b not available.

## Discussion

### Principal Findings

In a large, representative national survey, 70% of patients nationally viewed test results in their patient portal, and more than half (58%) viewed test results before discussing them with their HCP. However, only about a quarter (28%) indicated they were given the option to decide when to receive results or how these results were communicated to them. Rates of both viewing test results and being given the option to decide were lower among individuals with lower levels of education and digital literacy, which may be driven in part by differences in patient portal access more broadly [20], as was self-reported understanding of test results viewed before a discussion with their HCP. Only two-thirds of patients nationally reported they understood results they viewed before hearing from a provider and what those results meant for their care, which was lower than patient reports of general understanding of information in the patient portal and HCP explanations during health care visits (89%, respectively).

Patients who reported being encouraged to use their patient portal by their HCP were more likely to view immediately released results and indicate they understood what the results showed and what they meant for their care, which suggests anticipatory guidance from HCPs may help set expectations before results are delivered [21,22]. Together, these findings indicate that patients overall are highly engaged with results released immediately via the patient portal, but there may be missed opportunities to address differences in patient preferences around notification of newly released results.

In response to the information blocking regulations, many HCPs provided near-immediate availability of information in the patient record and portal as their organizational default, when technological capabilities allow [23]. The high rates of access to that information shown here, coupled with evidence on patient views from prior studies, suggest the importance of maintaining patients’ ability to engage with their health data [15,23,24]. At the same time, the information blocking regulations allow HCPs to grant a patient’s request to delay the release of their test results and allow patients to choose whether to receive notifications (eg, text messages) when results are available [22,25]. However, anecdotally, we have found that most vendor systems do not routinely offer functionality that allows patients to specify their preferred timing for the release of specific test results without custom configuration, which may limit opportunities for patients to indicate their preferences around the timing of result release in their patient portal.

Our findings indicate that while most patients nationally viewed immediately released results, only one-third reported being given the option to decide whether to receive results before discussing with their HCP. Interestingly, patients with a recent diagnosis of cancer were no more likely to indicate that they had been given an opportunity to decide when to receive results despite widespread concerns over the potential negative impact news of a cancer diagnosis might have on patient well-being [26,27], which has already led some states to propose legislation delaying the release of cancer results [28,29]. These findings speak to potential missed opportunities to reduce worry by offering options on how patients would like to receive results. HCPs, with support from system developers, could help address this shortcoming by making patients aware of options provided through the patient portal to indicate their preferences around the notification or release of results, through precounseling with patients during health care visits or regular communication via the portal, and through discussions with support staff [30].

Fewer patients indicated they understood test results they viewed before discussing with their HCP compared to the share of patients who indicated their HCPs explained information clearly during health care visits and that stated it was easy to understand information in their patient portal. These findings suggest test results may be more difficult to interpret or contextualize [31]. Consistent with prior work [32], patients with lower levels of digital literacy across self-reported measures were even less likely to report that they understood test results they viewed before discussing them with their HCP, which could lead to undue anxiety or worry about test results delivered without context [7]. Patients who reported being encouraged by their HCP to use their patient portal were also more likely to report being given the option to decide how to receive test results and to indicate they understood results viewed in their portal before discussing with their HCP, which suggests discussions about the patient portal may present an opportunity to discuss communication preferences or what different results would mean for subsequent care. There are likely many options to improve patient understanding of their results: discussions with HCPs prior to the availability of test results, clear handouts preparing patients for results, displaying the result in the context of a standard range, including links to definitions of medical terms, or even providing artificial intelligence-based tools to answer questions and alleviate burden on HCPs [22,33-36]. Each of these strategies merits investigation as health care moves toward a more digital and asynchronous delivery of care. Caregiver or proxy-user perspectives may also represent an important future research area, as these users often engage with patient portals and make decisions on behalf of patients [6,19,37].

### Limitations

This study has several limitations. First, survey data is self-reported and is therefore subject to recall bias. We tried to mitigate this source of bias by limiting analyses to respondents who had test results in the past year. To the extent that recall bias persists, it may indicate that current strategies related to the provision of options on when to receive test results are not sufficient to lead to informed decision-making by patients and leave a lasting impression. Second, HCP conversations as well as patient preferences regarding how and when information is communicated to them may depend on the sensitivity of the results. In this study, we were not able to distinguish between normal and abnormal test results, and whether this affected communication preferences or being given the option to decide how to receive results. However, we observed no difference between patients with a recent cancer diagnosis and others. Further, previous work suggests preferences for immediate release persist among patients with abnormal results, even if access is associated with increased worry, and that patient worry around test results may be driven by individual attitudes and preferences more so than the test itself [9,38]. Third, our measure of patients’ ease of understanding portal information may be influenced by their ability to review test results – the most common use of patient portals [19]. This may make it difficult to separate general comprehension of portal content from reactions to viewing results. Finally, our measure of patients’ understanding of results viewed before discussing with an HCP was subjective and may represent an overestimate of patients’ understanding of what results show and what they meant for their care. Prior work has shown that viewing immediately released test results was associated with a near doubling of messages patients send to their clinician, which suggests immediate access to test results does not diminish—and conversely, may increase—the need for HCPs to help interpret these results and what they mean for their patients’ care [23].

### Conclusions

In 2024, most patients who received test results reported viewing those results in their patient portal before hearing from their HCP, signaling a strong revealed preference for immediate release of test results. Only one-third of patients reported being given the option to decide how results would be communicated to them, indicating missed opportunities to avoid worry for those who did not want to see results before discussing with their HCP. Two-thirds of patients who viewed immediately released results indicated they understood what the results showed and what they meant for their care—substantially less than indicated other forms of communication were clear. In response to the information blocking regulations, many HCPs released test results to patients immediately upon their availability as the organizational default, and our findings suggest that, consistent with prior studies, patients prefer to access their results as soon as they are available. However, incorporating patient communication preferences and improving how results are presented in the portal may help to avoid undue worry about sensitive results while ensuring that patients have the tools and resources to access their health information and make informed decisions about their health and care.

## Supplementary material

10.2196/94098 Multimedia Appendix 1 Sample characteristics, survey questions to create key measures, and additional results.

## Abbreviations

HCP: health care provider
HINTS: Health Information National Trends Survey
NIH: National Institutes of Health
