# Supplementary material for: Patient-Reported Experiences With Viewing and Understanding Test Results in Patient Portals: Cross-Sectional Survey Analysis
Source: J Med Internet Res. 2026 Jun 12;28:e94098. doi: 10.2196/94098 (PMC13263015; doi:10.2196/94098)
Supplement: Multimedia Appendix 1 [file jmir-v28-e94098-s001.docx]

**Table S1**. Sample characteristics.

|  | All individuals (*N* = 6045) | | Portal users (*N* = 4485) | |
| --- | --- | --- | --- | --- |
|  | *N* | Weighted % | *N* | Weighted % |
| Age |  |  |  |  |
| 18-34 | 918 | 23% | 725 | 22% |
| 35-49 | 1,219 | 26% | 1,003 | 28% |
| 50-64 | 1,614 | 28% | 1,208 | 28% |
| 65-74 | 1,358 | 14% | 975 | 14% |
| 75+ | 936 | 9% | 574 | 8% |
| Education |  |  |  |  |
| Less than high school | 344 | 6% | 154 | 4% |
| High school | 935 | 20% | 535 | 17% |
| Some college | 1,791 | 39% | 1,295 | 39% |
| College | 2,975 | 35% | 2,501 | 40% |
| Digital literacy |  |  |  |  |
| 1 (low) | 2,280 | 33% | 1,356 | 27% |
| 2 | 813 | 13% | 615 | 12% |
| 3 | 1,836 | 33% | 1,577 | 36% |
| 4 (high) | 1,103 | 21% | 933 | 24% |
| Chronic condition |  |  |  |  |
| Yes | 3,994 | 62% | 2,967 | 63% |
| No | 1,967 | 38% | 1,460 | 37% |
| Recent cancer diagnosis |  |  |  |  |
| Yes | 302 | 3% | 241 | 4% |
| No | 5,461 | 97% | 4,039 | 96% |
| Encouraged to use patient portal |  |  |  |  |
| Yes | 4,558 | 78% | 3,989 | 90% |
| No | 1,458 | 22% | 485 | 10% |

**Table S2**. HINTS survey questions used to create key measures.

| Study Measure | HINTS Survey Question |
| --- | --- |
| **Key outcomes measures** | |
| View results | E5. In the past 12 months, have you used your online medical record or patient portal to…   1. Look up test results?   **Response options**: Yes / No |
| View results before hearing from HCP | E10. In the past 12 months, did you look at test results made available to you through your online medical record or patient portal before hearing about the result from your health care provider?  **Response options**: Yes / No / I did not have any medical tests in the past 12 months (this group excluded from study) |
| Option to decide | E9. In the past 12 months, were you given the option to decide whether or not you wanted to receive test results before your health  care provider could discuss them with you?  **Response options**: Yes / No / Don’t know |
| Understand results viewed before hearing from HCP | E11. How well did you understand what the test results showed and what they meant for your care?  **Response options**: Very well / Well / Fairly Well / Poorly |
| **Comparable measures related to understanding information** | |
| Ease of understanding information in patient portal | E6. How easy or difficult was it to understand the health information in your online  medical record or patient portal?  **Response options**: Very easy / Somewhat easy / Somewhat difficult / Very difficult |
| HCPs explain information clearly during health care visits | C4. The following questions are about your communication with all doctors, nurses, or other health professionals you saw during the past 12 months. How often did they do each of the following?   1. Explain things in a way you could understand.....   **Response options**: Always / Usually / Sometimes / Never |
| **Individual characteristics** | |
| Age | R1. What is your age? ____ Years old |
| Education | R8. What is the highest grade or level of schooling you completed?  **Response options**: Less than 8 years / 8 through 11 years / 12 years or completed high school / Post high school training other than college (vocational or technical) / Some college / College graduate / Postgraduate |
| Digital literacy | B5. How much do you agree or disagree with the following statements?   1. I find learning how to use new technology frustrating....... 2. I can use applications/programs (like Zoom) on my cell phone or computer without asking someone for help....... 3. I have the skills to find the health information I need on the Internet.......   **Response options**: Strongly agree / Somewhat agree / Somewhat disagree / Strongly disagree |
| Chronic condition | I5. Has a doctor or other health professional ever told you that you had any of the  following medical conditions:   1. Diabetes or high blood sugar? 2. High blood pressure or hypertension? 3. A heart condition such as heart attack, angina, or congestive heart failure?. 4. Chronic lung disease, asthma, emphysema, or chronic bronchitis? 5. Depression or anxiety disorder?   **Response options**: Yes / No |
| Recent cancer diagnosis | Q1. Have you ever been diagnosed as having cancer? **Response options**: Yes / No  Q3. At what age were you first told that you had cancer? _____  (We subtracted this from age to identify recent diagnoses in the past 5 years). |
| Encouraged to use patient portal | E2. Have any of your health care providers, including doctors, nurses, or office staff ever encouraged you to use an online medical record or patient portal?  **Response options**: Yes / No |

**Table S3**. Patients’ understanding of test results compared to information in the patient portal and HCP explanations, 2024.

| **HCPs explain information clearly during health care visits?** | |  |  |  |
| --- | --- | --- | --- | --- |
|  | *N* | Weighted % | 95% CI | |
| Always | 3,236 | 57% | 0.545 | 0.595 |
| Usually | 1,774 | 32% | 0.300 | 0.349 |
| Sometimes | 481 | 9% | 0.078 | 0.112 |
| Never | 50 | 1% | 0.008 | 0.021 |
| Total | 5,541 | 100% |  |  |
| **Ease of understanding information in patient portal** | |  |  |  |
|  | *N* | Weighted % | 95% CI | |
| Very easy | 2,075 | 45% | 0.420 | 0.473 |
| Somewhat easy | 1,892 | 44% | 0.416 | 0.473 |
| Somewhat difficult | 435 | 10% | 0.085 | 0.119 |
| Very difficult | 64 | 1% | 0.006 | 0.012 |
| Total | 4,466 | 100% |  |  |
| **Understand results viewed before hearing from HCP** | |  |  |  |
|  | *N* | Weighted % | 95% CI | |
| Very well | 1,155 | 34% | 0.309 | 0.372 |
| Well | 1,096 | 32% | 0.290 | 0.351 |
| Fairly well | 970 | 27% | 0.244 | 0.301 |
| Poorly | 211 | 7% | 0.056 | 0.084 |
| Total | 3,432 | 100% |  |  |

**Table S4.** Differences in patients’ and portal users’ reports of viewing test results in their patient portal (overall and before hearing from their HCP), being given the option to decide how to receive test results, and understanding results viewed before hearing from their HCP, by digital literacy measures, 2024.

|  | Viewed test results in patient portal | | Viewed test results before hearing from HCP | | Option to decide how to receive results | | Understand results |
| --- | --- | --- | --- | --- | --- | --- | --- |
| I find learning how to use new technology frustrating | All patients | Portal users | All patients | Portal users | All patients | Portal users | Portal users |
| 1. Strongly agree | 46% | 88% | 42% | 69% | 26% | 36% | 50% |
| 1. Somewhat agree | 67% | 89% | 57% | 73% | 26% | 31% | 59% |
| 1. Somewhat disagree | 75% | 94% | 64% | 79% | 28% | 33% | 68% |
| 1. Strongly disagree | 79% | 95% | 67% | 79% | 31% | 35% | 74% |
| I can use applications/programs (like Zoom) on my cell phone or computer without asking someone for help | All patients | Portal users | All patients | Portal users | All patients | Portal users | Portal users |
| 1. Strongly disagree | 48% | 92% | 44% | 78% | 26% | 38% | 55% |
| 1. Somewhat disagree | 55% | 85% | 42% | 61% | 21% | 26% | 58% |
| 1. Somewhat agree | 66% | 91% | 54% | 72% | 26% | 33% | 55% |
| 1. Strongly agree | 79% | 94% | 69% | 79% | 30% | 34% | 72% |
| I have the skills to find the health information I need on the Internet | All individuals | Portal users | All individuals | Portal users | All individuals | Portal users | Portal users |
| 1. Strongly disagree | 35% | 87% | 35% | 74% | 26% | 44% | 56% |
| 1. Somewhat disagree | 57% | 85% | 41% | 57% | 22% | 25% | 37% |
| 1. Somewhat agree | 65% | 90% | 55% | 73% | 25% | 31% | 56% |
| 1. Strongly agree | 78% | 95% | 68% | 80% | 31% | 35% | 73% |

**Notes**: Individual digital literacy measures come from question B5 of HINTS 7. For the digital literacy index used in the main manuscript, questions B5b and B5c were re-coded so that they move in the same direction as B5a (where strongly agree = lower literacy, and strongly disagree = higher literacy). After re-coding, for all variables values of 1 indicate lower digital literacy and values of 4 indicate higher digital literacy. The index averages across these measures.
